# Identity Transitions of Tapetum Phases: Insights into Vesicular Dynamics and in Mortem Support During Pollen Maturation

**DOI:** 10.3390/plants14050749

**Published:** 2025-03-01

**Authors:** Gabriel Luis L. S. Moreira, Maria Eduarda P. Ferreira, Francisco S. Linhares

**Affiliations:** Laboratório de Biologia do Desenvolvimento e Estrutura Vegetal, Centro de Energia Nuclear na Agricultura, Universidade de São Paulo, Piracicaba 13400-970, SP, Brazil; gabluismoreira@usp.br (G.L.L.S.M.); maria.penaferreira@usp.br (M.E.P.F.)

**Keywords:** programmed cell death, auxin, anther development, transcription regulation, vesiculation

## Abstract

Flower development progresses through twelve distinct stages, meticulously regulated to optimize plant reproductive success. At stage 5, the initiation of anther development occurs, which is further categorized into 14 stages divided into two defined phases: phase 1, known as microsporogenesis, and phase 2, termed microgametogenesis—encompassing pollen maturation and anther dehiscence. The maturation of pollen grains must be temporally synchronized with anther dehiscence, with auxin serving as a pivotal spatio-temporal link between these processes, coordinating various aspects of anther development, including stamen elongation, anther dehiscence, and tapetum development. The tapetum, a secretory tissue adjacent to the meiocytes, is essential for nurturing developing pollen grains by secreting components of the pollen wall and ultimately undergoing programmed cell death (PCD). This review primarily focuses on microgametogenesis, the identity and function of the tapetum during the different progression phases, the role of vesicular signaling in delivering external components crucial for pollen grain maturation, and the distinctive process of PCD associated with these developmental processes.

## 1. Overview of Anther Development

Flower development holds significant importance, as it is essential for the successful perpetuation of plant species. From an agricultural perspective, this process directly influences the yield of crops, including fruits, legumes, and grains. The progression of flower development occurs through 12 well-defined stages, each tightly regulated to ensure optimal reproductive success [1]. The progression of flower development occurs through 12 well-defined stages, each tightly regulated to ensure optimal reproductive success [1].

The alternation of generations between haploid gametophytes and diploid sporophytes is the foundation of sexual reproduction. This cycle is defined by the transitions of meiosis and fertilization, which drive the shift between these generations. Carpels and anthers serve as the primary organs where meiosis takes place, along with various other processes that shape the entire developmental pathway of the germline. Unlike animals, which possess predetermined germlines, plants must generate their reproductive organs and cell types de novo from non-reproductive tissues. The ABCE model of flower development governs floral architecture by regulating the expression of specific combinations of at least four key factors, which orchestrate the formation of the four concentric whorls during organogenesis [2].

**Figure 1:**
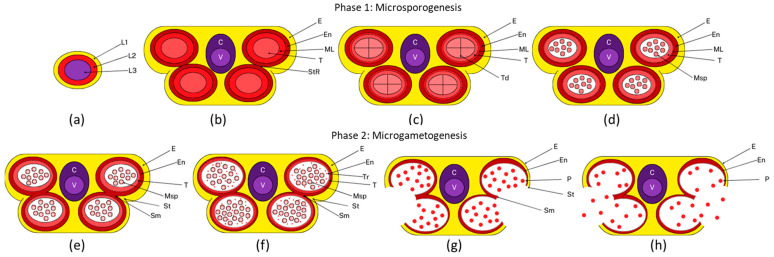
Schematic overview of anther development in *Arabidopsis thaliana*. Phase 1: (**a**) Anther development initiates at stage 1 with the differentiation of the L1, L2, and L3 cell layers within the stamen primordia. (**b**) By stage 4, all sporophytic tissues—epidermis (E), endothecium (En), middle layer (ML), tapetum (T), connective tissue (C), and vascular region (V)—are fully formed. (**c**) At stage 7, the gametophytic tissues have undergone mitosis and are encased within a callose wall, forming tetrads (Td). (**d**) By stage 8, the callose wall degrades, releasing microspores (MSp). Phase 2: (**e**) By stage 9, the middle layer has completely degenerated, and the microspores become vacuolated. (**f**) At stage 10, the tapetum begins to degenerate, with its remnants nourishing the developing microspores. (**g**) By stage 13, the pollen grains (PG) reach maturity, and degeneration of the stomium region (St) initiates anther dehiscence. (**h**) Stage 14 marks the release of mature pollen grains, accompanied by the shrinkage of the anther. Abbreviations: L1, L2, L3 (cell layers in stamen primordia); E (epidermis); En (endothecium); C (connective tissue); V (vascular region); ML (middle layer); T (tapetum); StR (stomium region); Td (tetrad); MSp (microspores); St (stomium); Sm (septum); PG (pollen grains).

Anther development starts at stage 5 of the flower development with the appearance of six stamen primordia, and is itself divided in 14 specific stages [3]. Two main phases are easily distinguishable: phase 1, termed microsporogenesis or early development (stages 1–8 of anther development) (Figure 1a–d); and phase 2, termed microgametogenesis or the maturation and senescence phase (stages 9–14 of anther development) (Figure 1e–h) [3]. During anther stages 1 to 5 (corresponding to floral stages 5 to 9), divisions in the L1 cell layer establish the stamen epidermis, while divisions in the L3 layer generate connective tissue and vascular structures (Figure 1a,b). Concurrently, periclinal divisions of L2 cells, known as archesporial cells, produce primary parietal and primary sporogenous cells, which subsequently develop into the four radially symmetrical microsporangia (Figure 1a,b). In most angiosperms, four distinct somatic cell layers encase the germinal cells at the onset of meiosis, arranged from the outermost to innermost as follows: the epidermis, endothecium, middle layer, and tapetum (Figure 1c). Structurally, anthers exhibit bilateral symmetry, consisting of two thecae, each comprising an abaxial (lower) and adaxial (upper) lobe. Each lobe functions as a microsporangium, providing a protective environment for the developing germinal cells. The four lobes of the anther are united by the connective tissue, which encases the central vascular tissue. The connective and vascular columns extend along the *y*-axis of the anther, continuing into the filament that anchors the anther to the base of the flower.

The initial stages of anther development (stages 1–4) are characterized by cell division, specification, and differentiation leading to the formation of sporophytic tissues, such as epidermis, endothecium, middle layer, tapetum, connective tissue, and vascular bundle. In *Arabidopsis*, a mature anther consists of pollen sacs and connective tissues. During stage 2, four hypodermal cells located at each of the four corners of the developing anther expand radially and differentiate into an archesporial cell [3,4]. At stage 3, these cells undergo periclinal divisions to form an outer layer and an inner layer, i.e., primary parietal cells (PPCs) and primary sporogenous cells (PSCs), respectively. Concurrently, specification of the regions that will give rise to the filament occurs and the anther become distinct. PPCs divide both anticlinally and periclinally to form sporophytic tissues, while PSCs develop into microsporocytes. Experimental data indicate that auxin, produced by *YUC* genes in juvenile floral buds, is initially translocated directionally towards the apex of the primordium. At this location, auxin attains a critical concentration necessary for the formation of stamen primordia. Subsequently, it is transported basipetally to the interior of the floral structure [5]. By stage 5, the gametophytic phase begins, with PSCs developing into microspore mother cells (MMCs). At stage 6, MMCs undergo meiotic divisions, distinguished by a unique single cytokinesis, which results in the formation of tetrads enclosed by callose, a structure evident by stage 7 (Figure 1c) [3]. Nonetheless, the normal development of the tapetum appears to be essential for the completion of the male meiotic cell cycle. Recent experiments indicate that small RNAs (sRNAs) found in the anthers, including microRNAs (miRNAs) and phased secondary small interfering RNAs (phasiRNAs), may play a significant role in regulating male meiosis [6]. The degeneration of the middle layer and the vesiculation of tapetal cells begin as early as stages 6 to 8 [3,7]; however, recent findings indicate that this tissue continue to coexist, albeit marginally, until stage 11 [8] (Figure 1). By stage 8, callose degradation leads to the release of microspores from the tetrads followed by their first mitotic division, marking the conclusion of the first developmental phase (Figure 1d).

Phase 2 is defined by the differentiation and maturation of pollen grains. At stage 9 (Figure 1e), tapetal cells become mainly vacuolated and accumulate vesicle bodies, such as tapetosomes (originating from the endoplasmic reticulum) and elaioplasts (derived from proplastids). By stage 10 (Figure 1f), these cells initiate PCD, releasing remnants and vesicles into the locules. Following their release from callose walls at stage 8, pollen grains undergo a mitotic division by stage 11. At this stage, no remnants of the tapetum are detectable, and the pollen grains display mature ornamentation. Additionally, during stage 11 the process of dehiscence begins, consisting in the expansion of the endothecium, accompanied by subsequent deposition of ligno-cellulosic thickening in both endothecium and connective cells. By stage 12, pollen grains become trinucleated, while the septum undergoes degeneration and breakage, resulting in the formation of bilocular anthers [3]. Stage 13 involves complete anther dehiscence, characterized by the opening of the stomium (Figure 1g). This is driven by tensional forces of a lignified and dehydrated endothecium, along with anther growth, ultimately leading to the release of fully mature pollen grains at stage 14 (Figure 1h) [9].

Pollen development in rice seems to proceed through a developmental pathway analogous to that seen in *Arabidopsis*, involving the formation of a secretory tapetum [10,11].

## 2. The Tapetum Nourishes Pollen Wall

**Figure 2:**
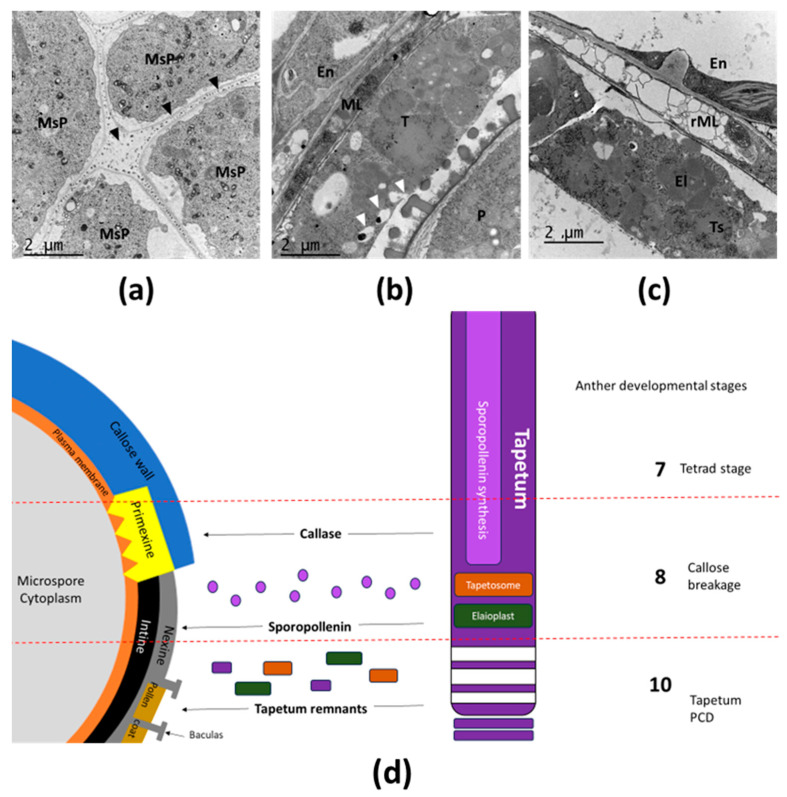
Different stages of tapetum secretion. (**a**) Non-vesicular secretion of tapetum (black arrows) along the callose wall that form the tetrads of microspores by stage 7 of anther development. The constituents of primexine are secreted by the tapetum during the tetrad stage. In addition, tapetum secretes callase which facilitates the breakdown of the callose wall, resulting in the release of microspores. (**b**) Vesicular secretion of the tapetum (white arrows) supports the ornamentation of the pollen wall. Throughout pollen development, the tapetum exhibits significant vesicular activity, supplying the pollen wall with sporopollenin components. (**c**) Mature tapetossomes and elaioplasts are formed during the tapetum PCD. Tapetosomes and elaioplasts are vesicles rich in wax and proteins that are released following PCD, contributing to the pollen coat. Abbreviations: MsP (microspore), En (endothecium), ML (middle layer), T (tapetum), P (pollen grain), rML (residual of middle layer). (**d**) Current model of pollen wall formation. During the tetrad stage (stage 7), microspores are enclosed within a callose wall. At this stage, the plasma membrane begins to exhibit an undulating structure, creating an interspace for the formation of the primexine layer. Following the enzymatic degradation of the callose wall by callase at stage 8, the primexine acts as a receptor for sporopollenin precursors. These sporopollenin precursors, secreted by the tapetum, serve as the foundation for exine development. As sporopollenin polymerizes, the exine enlarges and differentiates into sublayers, including the intine and nexine. By stage 10, programmed cell death (PCD) of the tapetum releases remnants such as tapetosomes and elaioplasts, which are deposited onto the outer surface of pollen grains. These deposits fill the cavities of the nexine, ultimately forming the pollen coat.

Pollen grain maturation is characterized by the formation of a complex pollen wall, which is preceded by the sequential deposition of multiple transient extracellular layers on the surface of the developing pollen grains (Figure 2). At first, a thin ephemera structure called primexine is formed between plasma membrane and callose wall. Primexine is a highly dynamic layer that undergoes changes in its xylan and pectin composition, along with modifications to its ultrastructure, throughout the tetrad stage of development. Recently, it has been shown that NEW ENHANCER OF ROOT DWARFISM 1 (NERD1), a trans-membrane protein localized in the Golgi of tapetum and microspores, is essential for the plasma membrane undulation, which is crucial for proper primexine deposition [12]. In the late tetrad stage, primexine creates a scaffolding pattern for formation of reticulate pollen wall [13]. The pollen wall is primarily composed of sporopollenin, which is secreted by the tapetum. The exine layer of the pollen wall features a complex pattern and is predominantly made of sporopollenin, a durable polymer consisting of fatty acids and phenolic compounds. Tapetal cells synthesize the precursors of sporopollenin through the action of various enzymes, and these precursors are subsequently transported to the surface of developing pollen grains. Although the mechanisms underlying this transport remain poorly understood, the *Arabidopsis* ABC transporter ABCG26/WBC27 has been identified as playing a crucial role in mediating the transport of sporopollenin precursors [14]. Structurally, the exine is organized into distinct sublayers: the inner intine, the nexine, and the outermost sculptured layer known as the sexine, which forms baculae and tectum. The cavities within the sexine are filled with the pollen coat, which is derived from the remnants of tapetal cells that have undergone PCD. This pollen coat, rich in proteins, lipids, and pigments, is essential for facilitating pollen–stigma interactions [15]. Upon reaching maturity, the PCD of the septum and stomium signals the onset of anther dehiscence, leading to the release of pollen grains [3]. The tapetum has recently been identified as a model tissue for secretory processes in plant cells [16], due to its intense intracellular and exocytic trafficking, and it particularly presents different secreting stages as non-vesicular (Figure 2a) and vesicular secretion (Figure 2b), as by the release of its remnants as tapetosomes and elaioplasts by its own PCD (Figure 2c), synthetized at Figure 2d.

The maturation of pollen grains must be precisely coordinated with anther dehiscence and filament elongation. Consequently, tapetal development, which provides essential components for pollen maturation, must synchronize with the development of the endothecium until its degeneration. Auxin likely serves as a key link, coordinating the spatial and temporal aspects of pollen maturation, anther dehiscence, and filament elongation. It has been shown that auxin is synthesized in pre-meiotic stages of meiocytes by YUC2 and YUC6, where *PIN5* and *PIN8* are expressed [17]. Both auxin biosynthesis genes are also active from stage 8 of anther development in the tapetum, but at stage 8, auxin accumulates only in the middle layer probably due to auxin transport via ABCB19/PGP19 [18,19]. Chromatin immunoprecipitation (ChIP) and electrophoretic mobility shift assays (EMSA) have shown that ARF17 can directly bind to the *CalS5* promoter. Although *cal5* mutants produce a reduced amount of primexine, they are still capable of generating viable seeds [19,20]. In contrast, *arf17* mutants are completely sterile, displaying an absence of primexine deposition. Additionally, various isoforms of ARF8 (auxin response factor 8) regulate different aspects of anther development [21,22]. For example, the splice variants ARF8.2 and ARF8.4 are involved in stamen elongation and anther dehiscence, respectively [21], while ARF8.1 specifically regulates pollen ornamentation by controlling the expression of transcription factors (TFs) within the tapetal developmental network [22]. The next section aims to explore these TFs and their roles throughout tapetal development, culminating in its PCD.

## 3. Tapetal Development: From Specification to Specialized Nurturing Throughout Different Anther Stages, Culminating with Its Programmed Cell Death (PCD)

After flower meristem transition is established, different whorls emerge by the means of specific flower identity genes, and stamen are identified by *APETALA 3* (*AP3*), *PISTILLATA* (*PI*), *AGAMOUS* (*AG*), and *SEPALLATA 1/2/3/4* (*SEP1/2/3/4*) in the third floral whorl. During microsporogenesis, the L2 layer, identified by expression of the MADS-box TF *SPOROCYTELESS/NOZZLE* (*SPL/NZZ*), gives rise to archesporial cells, that originate primary parietal cells. The tapetum arises from inner parietal cells and is specified by a signaling network constituted by the complex of TAPETUM DETERMINANT 1 (TPD1), EXCESS MICROSPOROCYTES1/EXTRA SPOROGENOUS CELLS (EMS1/EXS), and SOMATIC EMBRYOGENESIS RECEPTOR-LIKE KINASE 1/2 (SERK1/2). In the complex, EMS1 seems to recognize TPD1, with SERK1/2 acting as co-receptors [23]. The initial stages of pollen development in rice exhibit a strong resemblance to those in *Arabidopsis*. The gene *MULTIPLE SPOROCYTE1* (*MSP1*), encoding a leucine-rich repeat (LRR) receptor-like kinase, appears to be the orthologue of the *Arabidopsis EMS1/EXS* gene.

**Figure 3:**
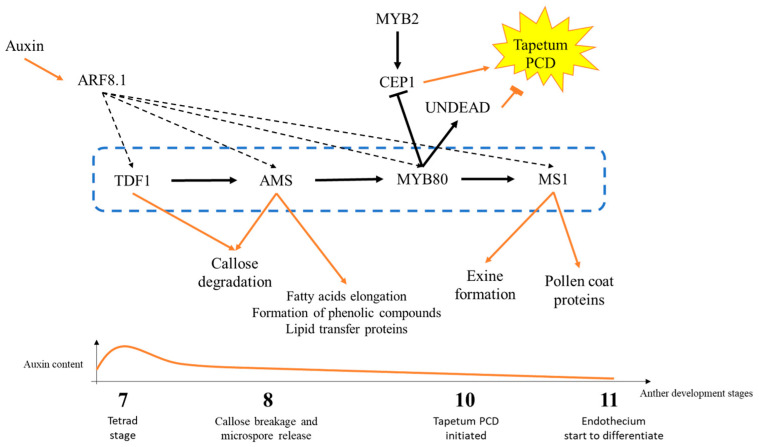
TFs involved in tapetum function and its development. During anther development, auxin exhibits a peak concentration in the middle layer during the microspore stage, after which its levels gradually decrease until endothecium differentiation (stage 11) which starts the process of anther dehiscence. ARF8.1, a TF sensitive to auxin, directly binds to the promoters of *TDF1*, *AMS*, *MYB80*, and *MS1*. TDF1 and AMS (downstream of TDF1) are involved in callase secretion thus promoting the release of microspores (stage 8). AMS is considered the master regulator of tapetum vesiculation, influencing fatty acids elongation, the formation of phenolic compounds and the synthesis of lipid transfer proteins. Downstream of AMS, MYB80 regulates the tapetum PCD (stage 10) through the regulation of *CEP1* and *UNDEAD* transcription. CEP1 and UNDEAD are proteases associated to the tapetum PCD, where UNDEAD has been shown to inhibit tapetum PCD, while CEP1 seems to promote it. *CEP1* is upregulated by MYB2, which is a key TF in promoting tapetal PCD. Downstream of MYB80, MS1 promotes pollen grain ornamentation through promoting the exine and pollen coat formation. Blue dashed box: TF network of tapetum development; black solid arrows: transcriptional regulation; black dashed arrows: transcription regulation by ARF8.1; red solid arrows: effects of the TFs in tapetum function and development.

Tapetal cells are regulated by a cascade regulatory network of TFs that establish transition points of tapetal functioning throughout different stages. Consequently, the tapetum performs diverse functions and nurturing roles, commencing with the degradation of callose walls, progressing to the formation of the exine via exocytosis, and culminating in the deposition of the pollen coat through PCD. These TFs are DYT1, TDF1, AMS, MYB80, and MS1 (Figure 3). The most upstream TF during tapetal development is a bHLH TF, DYSFUNCTIONAL TAPETUM1 (DYT1) [24], directly binding upstream of the R2R3 MYB TF family, TAPETAL DEVELOPMENT and FUNCTION1 (TDF1) [25]. Both *dyt1* and *tdf1* exhibit impaired development of the tapetum and consequently male sterility, with *tdf1* showing callose degradation defects, as reported by aniline staining [26].

These defects seem also to be related with the low expression of *ABORTED MICROSPORES* (*AMS*) in *tdf1*. Recent reports have shown that TDF1 directly regulates the expression of *AMS* via an AACCT cis-element [27] (Figure 3). AMS is a bHLH TF that promotes the expression of more than 70 genes involved in several processes of tapetum secretion, such as callose dissolution, fatty acids elongation, formation of phenolic compounds, and lipid transport [28], which also has a putative ortholog in rice, TDR [29]. Moreover, AMS is also involved in tapetum PCD through promoting the transcription of *MALE STERILE188/MYB80/MYB103* (*MYB80*) [30] (Figure 3). In rice, *tdr* mutant showed significant alterations in the aliphatic composition of anthers, with reductions in fatty acids, primary alcohols, alkanes, and alkenes. There was a notable increase in secondary alcohols with carbon chain lengths from C29 to C35. Microarray data indicated that genes related to lipid transport and metabolism were significantly altered in the *tdr* mutant, underscoring the importance of TDR in pollen wall formation. Additionally, 236 genes exhibited significant expression changes compared to the wild type, with 154 genes upregulated and 82 genes downregulated [31].

Recently, it has been demonstrated that TDF1-regulated reactive oxygen species (ROS) homeostasis plays a significant role in inhibiting lateral cell division and promoting cell differentiation in the tapetum. Specifically, the *sks18* (*SKEWED5-SIMILAR18*) knockout mutant, which is a downstream target of TDF1 and encodes a multicopper oxidase-like protein with ascorbate oxidase activity, exhibits low accumulation of ROS and additional layers of tapetal cells. Similarly, the overexpression of *VITAMIN C DEFECTIVE1* (*VTC1*), which is repressed by TDF1, also results in the presence of extra tapetal cells due to elevated ascorbate (AsA) concentrations [32].

MYB80 is a TF that regulates tapetal PCD by downregulating the expression of *CEP1* and upregulating the expression of *UNDEAD* [33] (Figure 3). Both UNDEAD and CEP1 are proteases that antagonistically influence tapetal PCD. In *undead* knock-down mutants, there is evidence of precocious tapetal PCD [34]. Conversely, while *CEP1* overexpression results in a similar phenotype, *cep1* mutants exhibit disrupted tapetal PCD [32] (Figure 3). This indicates that UNDEAD regulates the initiation of tapetal PCD, whereas CEP1 facilitates its progression. Indeed, CEP1 has been identified as a key executor of tapetal PCD [35]. In addition, *CEP1* is transcriptionally upregulated by MYB2, another important transcription factor involved in tapetal PCD [36] (Figure 3).

Furthermore, MYB80 also plays a role in tapetal vesiculation, as it directly regulates the expression of *MS1* [37] (Figure 3). The knockout *ms1* mutant exhibits abnormal tapetal secretion and disrupted exine formation, and no signs of programmed cell death (PCD). Instead, the presence of large autophagic vacuoles and mitochondrial swelling indicates that a necrotic-based breakdown of the tapetum is occurring, rather than the normal, regulated PCD process. Furthermore, TUNEL analysis failed to identify DNA fragmentation in the *ms1* tapetum [38]. Downstream of MS1, genes that encode pollen coat proteins, such as *GRP14*, *GRP17*, *GRP18*, *GRP19*, *EXL4*, and *EXL6*, are translated in the tapetum and accumulate later in the anther locule after tapetal PCD [37]. Additionally, in *ms1* mutants, tapetal cells fail to undergo normal PCD [38]. Interestingly, a recent study [22] had demonstrated that *TDF1*, *AMS*, *MYB80*, and *MS1* are all directly regulated by auxin through the splice variant ARF8.1 (Figure 3). During anther development, auxin levels peak in the middle layer during the microspore stage, gradually decreasing until endothecium lignification [39]. Thus, it appears that auxin mediates the coordination of anther development by aligning the maturation of pollen grains with the dehiscence of the anther. Given that the middle layer is situated physically between the tapetum and endothecium, it may be responsible for emitting signals that facilitate the spatio-temporal coordination of the onset of both developmental processes.

In short, the TFs network driving tapetum development is a cascade that ends in tapetum PCD. Nonetheless, all knockout mutants, including *ams*, *myb80*, and *ms1*, exhibit defects in tapetal PCD and, consequently, in pollen ornamentation. However, defects in pollen ornamentation are not exclusively related to imbalances in tapetal PCD. Pollen ornamentation can also be affected by impairments in the transport of materials from the tapetal cells to the developing pollen grains. Therefore, TDF1, AMS, MYB80, and MS1 are TFs that play a role not only in tapetal development but also in its secretory functions. For instance, TDF1 and AMS1 are involved in the secretion of the callase, which ultimately facilitates the breakdown of the callose wall surrounding developing microspores [26,27].

## 4. Tapetal Vesicle Formation and Cargo Sorting

Before PCD occurs, the tapetum reaches its maturity and exhibits a high content of tapetosomes (vesicles derived from the endoplasmic reticulum) and elaioplasts (vesicles derived from plastids). Subsequently, during tapetal PCD, these vesicles are released and deposited onto the external surface of developing pollen grains [19].

AMS has been proposed as the master regulator of communication between the tapetum and developing pollen grains, positively regulating 70 genes, including 23 that are involved in tapetal secretory functions such as callose degradation, fatty acid elongation, phenolic compound production, and lipid transport [28]. AMS regulates the expression of *TEK*, which is highly expressed during the tetrad stage [40]. *TEK* encodes a AT-hook nuclear-localized protein that negatively regulates the expression of *CalS5* [40]. CalS5 is required for callose synthesis and must therefore be inhibited by the end of the tetrad stage. In contrast, TEK promotes the expression of *AGP6*, *AGP11*, *AGP12*, and *AGP40*, which encode Arabinogalactan proteins responsible for nexine formation [40]. Thus, while *TEK* is expressed in the tapetum, its effects are observable during pollen development through its regulation of callose deposition and, subsequently, pollen wall deposition.

TEK is a TF that operates downstream of AMS, as previously demonstrated by several studies [40]. In addition, AMS also promotes the expression of various protein families, including transporters such as ABCG26, an adenosine triphosphate-binding cassette (ABC) transporter. ABCG26 is localized at the plasma membrane and is involved in the transport of sporopollenin precursors [14]. Another gene regulated by AMS, *IMPERFECTIVE EXINE FORMATION* (*IEF*), encodes a plasma membrane protein. *ief* knockout mutants exhibit defective formation of both nexine and exine [21]. Therefore, it is hypothesized that IEF may function as a transporter of sporopollenin precursors, similar to ABCG26. Additionally, several other transporters are involved in tapetal trafficking.

In light of the positive feedback regulatory loops between DYT1 and its downstream basic helix-loop-helix (bHLH) transcription factors, it is essential to have a pathway to prevent excessive overexpression of the tapetal transcriptional network and to maintain normal functional levels. In this sense, the peptide signal CLAVATA3/EMBRYO SURROUNDING REGION-RELATED 19 (CLE19) and some of its functionally redundant CLE family members have been found to prevent overexpression of the tapetum transcriptional network and ensure normal functionality, by limiting the expression of *AMS*. CLE19 directly interacts with the ectodomain of PXY-LIKE1 (PXL1) inducing its phosphorylation. Additionally, PXL1 is necessary for the action of CLE19 via SERK interaction. They are suggested to act as receptor and coreceptor, respectively, thereby regulating tapetum gene expression and pollen development [41].

Adaptor proteins (APs) are heterotetrameric complexes that play a crucial role in the recruitment of cargo and coat proteins during vesicle formation. AP-1 associates with the trans-Golgi network/early endosomes (TGN/EE), which serves as the central protein sorting station in plant cells [42]. For instance, ABCG9 and ABCG16 are two plasma membrane transporters that facilitate transport from the tapetum to the pollen surface. In knockout mutants of AP1/1β adaptins, ABCG9 and ABCG16 were mislocalized to the trans-Golgi network [16]. The AP-1µ/HAPLESS13 (AP1/HAP13) complex is essential for pollen sac formation and the sporophytic regulation of pollen production. The functional loss of HAP13 leads to a reduction in the number of pollen sacs and disrupts the tapetum’s function, compromising both its secretory activity during early developmental stages and its PCD at later stages. Additionally, *hap13* anthers display disruption in the polar distribution of auxin maxima via an asymmetric distribution of PIN1 [43].

Phosphoinositides, while regulating various processes in vesicular trafficking, also appear to play a significant role in PCD. In the roots of *Arabidopsis thaliana*, the enhancement of phosphatidylinositol 4,5-bis-phosphate (PI4,5P2) promotes early PCD and subsequently facilitates the elaboration of secondary cell walls in xylem cells [42]. The homeostasis of PI4,5P2 impacts cell trafficking toward the vacuole, and its rupture promotes protoxylem differentiation [44]. However, little is known about the effects of phosphoinositides on tapetal PCD, likely due to a lack of research on the function of these components in aerial organs. Notably, a significant study by Noack et al. (2022) [45] demonstrated the role of PI4Kα1 in tapetal function. Knockout mutants *pi4kα1* exhibit defects in the intine layer on the surface of pollen grains, indicating a role for phosphoinositides in the communication between the tapetum and pollen grains.

Other membrane components also play a role in both tapetal PCD and vacuolar trafficking. *MON1* (*MONENSIN SENSITIVITY1)* is highly expressed in the tapetum and encodes a protein that activates Rab7 [46]. RAB7 is a RAB GTPase protein that resides in membrane compartments, facilitating their trafficking to the vacuole [47]. Through the activation of RAB7, MON1 mediates the transport of CYS proteases from pre-vacuolar compartments to vacuoles, where these proteases are matured. CYS proteases are essential for tapetal PCD and accumulate in pre-vacuolar compartments in mon1 knockout mutants.

Furthermore, the tapetal cells in *mon1* delays PCD, which further compromises the pollen coat formation. Dysfunction in vacuole biogenesis, associated to changes in vacuole size, is associated with the dysregulation of tapetal PCD. This effect is evident in *mon1* plants as well as in all mutants deficient in key transcription factors involved in tapetal development, including *dyt1*, *tdf1*, *ams*, *myb80*, and *ms1* [26,36,48,49]. Interestingly, larger vacuoles and delayed PCD are also observed in *arf8-7* mutants that lack the expression of *ARF8.1* [22]. These findings suggest that auxin homeostasis can influence tapetal development and its PCD by affecting vesicular trafficking.

In conclusion, the proper localization of membrane proteins is essential for their function, relying on the action of other membrane-associated proteins, such as AP-complexes, as well as the lipid composition of the membrane (for example, the concentration of phosphoinositides that attract polybasic proteins). In this context, vesicular trafficking between organelles is particularly important for tapetum development.

## 5. Environmental Cues Affecting Tapetal PCD

**Figure 4:**
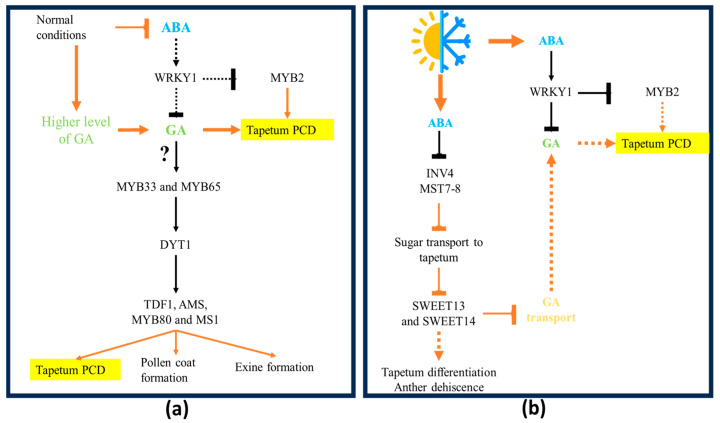
Effects of environmental stress on tapetum PCD. (**a**) Under normal (non-stress) conditions, levels of abscisic acid (ABA) in the tapetum are low, while gibberellin (GA) levels are elevated. High GA levels induce the transcription of *MYB33* and *MYB65*, which in turn upregulate transcription factors (TFs) essential for tapetum development and the timely progression of PCD. (**b**) During abiotic stress conditions, ABA accumulates to high levels within the tapetum, triggering the upregulation of *WRKY1*, a transcription factor that represses the expression of *MYB2*, a key regulator of tapetum PCD. WRKY1 also downregulates genes involved in GA biosynthesis. Furthermore, ABA interferes with sugar transport to the tapetum by downregulating *INV4* and *MST7-8*. This reduction in sugar transport suppresses the expression of *SWEET13* and *SWEET14*, resulting in decreased GA transport and further disruption of tapetum PCD. Legend keys: solid orange arrows indicate physiological effects, while dashed orange arrows denote physiological effects that are suppressed. Solid black arrows represent transcriptional regulation, and dashed black arrows indicate inhibited transcriptional regulation. A question mark indicates a known indirect relationship with no available information about the intermediating factors.

PCD in plants plays a crucial role in development, serving as a mechanism for nurturing and ornamenting newly formed cells from the outside [50]. Additionally, plant PCD acts upon exogenous cues as part of response mechanisms to herbivory, pathogen attacks, and abnormal environmental changes such as drought and thermal stresses [50]. Consequently, PCD can be categorized into developmental PCD (dPCD) and environmental PCD (ePCD), which are governed by distinct regulatory networks [51,52]. Although tapetal PCD is primarily a dPCD process, it can also be affected by environmental cues through an imbalance in hormone homeostasis [53,54]. Abscisic acid (ABA) has been identified as a key hormone influencing tapetal PCD in response to environmental signals [55] (Figure 4). During drought stress, *Arabidopsis* anthers exhibit upregulation of genes associated with ABA signaling (Figure 4b). Sustained water stress for 80 h, elevates ABA levels within the anthers while simultaneously delaying tapetal PCD [55]. Under normal conditions, ABA levels in the anther are at their lowest during tapetum PCD [56] (Figure 4a). Furthermore, the overexpression of ABA biosynthetic genes in rice spikelets suppresses tapetal PCD [57]. However, the mechanisms through which ABA influences tapetal PCD require further investigation for a comprehensive understanding. This mechanism may be elucidated by the relationship between ABA and MYB, a key transcription factor (TF) regulating tapetal PCD [33]. MYB2 also functions as a transcriptional activator of ABA signaling [58]. During ABA treatment, *MYB2* is repressed by WRKY1, a well-documented TF that repress gibberellin biosynthesis (GA) [59].

It is well established that ABA and GA exert antagonistic effect regulating the tapetum PCD under stress conditions [53,54] (Figure 4b). While ABA concentrations in flowers tend to increase during stress conditions, GA levels typically decrease [50]. Furthermore, ABA influences GA transport in the tapetum through its effects on anther sugar metabolism [53].

Exposure to low temperatures under 16 °C during the reproductive stage disrupts meiosis, tapetal programmed cell death (PCD), pollen viability, and fertilization, leading to plant sterility. The primary cause of pollen sterility under cold conditions is tapetal dysfunction, which impedes nutrient supply to developing microspores and disrupts their timely PCD. It is also well known that low temperatures decrease cell membrane fluidity and alter the conformation of membrane proteins [60,61,62], leading to a surge in reactive oxygen species (ROS). This results in induced membrane lipid peroxidation and increased levels of malondialdehyde, ultimately disrupting cell membrane structures, which are fundamental during PCD [63]. Under cold stress, elevated levels of ABA reduce the expression of genes involved in sugar metabolism, such as *INV4* (*INVERTASE 4*) and *MST7-8* (*MONOSACCHARIDE TRANSPORTER 7-8*) [64]. Invertases and sugar transporters facilitate the transport of sucrose from the epidermis and ultimately from the middle layer to the tapetum and developing microspores [65]. Additionally, sucrose and hexoses are transported from tapetum to developing pollen grains by sugar transporters such as SUT and SWEET proteins [53]. Notably, AtSWEET13 and AtSWEET14 also participate in GA transport. The double mutant *sweet13/sweet14* displays defects in anther dehiscence that can be rescued by GA application [66]. Furthermore, AtSWEET13 has been shown to regulate pollen wall formation [67].

In conclusion, ABA inhibits sugar transport into the tapetum during cold stress via INV4 and MST7-8, resulting in defects in tapetum differentiation and a subsequent delay in PCD. This imbalance in sugar metabolism not only depletes pollen starch content but also disrupts GA transport, further affecting anther dehiscence.

In contrast to the effects of ABA, increased levels of GAs promote tapetum differentiation and the initiation of its PCD [18]. In the *Arabidopsis* thaliana mutant *ga1-3*, which lacks GA signaling, the tapetum exhibits impaired PCD [16]. Additionally, MYB33 and MYB65 are transcription factors involved in tapetal development that are GA-sensitive. The double mutant *myb33/myb65* displays tapetal hypertrophy during the pollen meiotic cell stage, leading to pre-meiotic abortion of pollen development [68]. Therefore, MYB33 and MYB65 provide molecular evidence for the relationship between GA and tapetal PCD.

## 6. Conclusions and Future Perspectives

Anther development must be precisely regulated to ensure reproductive success. Dehiscence should occur neither too early nor too late, but rather at the appropriate time when pollen grains reach maturity. The middle layer tissue may play a key role in coordinating these developmental events, as it is positioned between the tapetum and endothecium. Notably, the middle layer is a transient tissue that influences the development of adjacent tissues. A peak in auxin concentration in the middle layer at stage 7 may initiate the temporal coordination of pollen maturation and anther dehiscence. Auxin regulates various stages of anther development through different splice variants of ARF8. However, molecular studies investigating the effects of impaired auxin synthesis, transport and/or signaling specifically in the middle layer are needed to demonstrate the timely coordination of pollen maturation and anther dehiscence associated with the auxin peak in this tissue.

The secretory function of the tapetum is regulated by the same TFs that govern its development. For example, mutants such as *tdf1*, *ams*, *myb80*, and *ms1* exhibit defects in vacuole biogenesis within the tapetum and in tapetal PCD. The complex relationship between vacuole function and exocytosis is still poorly understood. Moreover, almost no attention to the biogenesis and transport defects of tapetosomes and elaioplasts in these mutants has been given. These vesicles are formed in the tapetum prior to PCD and subsequently contribute to pollen coat formation. The gap in understanding the factors involved in the formation of tapetosomes and elaioplasts necessitates further investigation at the single-cell level. New single-cell techniques with high sensitivity could provide solutions to these questions. For example, single-cell transcriptomics, although not yet widely applied in plants, could elucidate the transcription factors involved at various stages of tapetal development. Additionally, while laser-induced breakdown spectroscopy (LIBS) has not been extensively used for single-cell analysis, it has successfully analyzed single pollen particles, demonstrating its potential for such applications [69]. Moreover, ablation inductively coupled plasma mass spectrometry (LA-ICP-MS) combines the laser ablation process with conventional ICP-MS, offering high sensitivity and simple histological tissue preparation, thus providing new insights into tapetal composition during different developmental stages [70].

## Data Availability

No new data were created or analyzed in this study.
